# Gut microbial communities associated with phenotypically divergent populations of the striped stem borer *Chilo suppressalis* (Walker, 1863)

**DOI:** 10.1038/s41598-021-94395-y

**Published:** 2021-07-22

**Authors:** Haiying Zhong, Juefeng Zhang, Fang Li, Jianming Chen

**Affiliations:** 1 Institute of Plant Protection and Microbiology, Zhejiang Academy of Agricultural Sciences, Hangzhou, 310021 China; 2 State Key Laboratory for Managing Biotic and Chemical Threats To the Quality and Safety of Agro-Products, Hangzhou, 310021 China

**Keywords:** Microbiology, Zoology

## Abstract

*Chilo suppressalis* (Walker, 1863) is a serious stem borer of rice and water-oat plants, and has phenotypically diverged into rice and water-oat populations. Insect gut microbiota plays an important role in the host life and understanding the dynamics of this complicated ecosystem may improve its biological control. The effect of diet and gut compartments on the gut microflora of divergent populations of *C. suppressalis* is not fully clear. Herein, we characterized the gut microbiota of *C. suppressalis* populations fed on two hosts (i.e., water-oats fruit pulps and rice seedlings), by sequencing the V3–V4 hypervariable region of the 16S rRNA gene using the Illumina MiSeq platform. Gut bacterial communities showed variation in relative abundance among *C. suppressalis* populations fed on water-oats fruit pulps or rice seedlings. Proteobacteria and Firmicutes became the predominant phyla, and Enterobacteriaceae, Enterococcaceae and Halomonadaceae were the predominant family in all *C. suppressalis* populations*.* The highest bacteria diversity was found in the midgut of the rice population fed on water-oat fruit pulps. Bacterial communities in the midgut were more diverse than those in the hindgut. The bacterial genera distribution showed great differences due to diet types and gut compartments among populations. Our results demonstrated that the host plants tested had a considerable impact on gut bacterial composition of *C. suppressalis* populations. Additionly, the unique gut morphology and physiological conditions (viz., oxygen content, enzymes) also contributed to variation in microbiomes. In conclusion, our study provided an important insight into investigation of insect-bacteria symbioses, and biocontrol of this species and other related lepidopterans.

## Introduction

*Chilo suppressalis* (Walker, 1863) is one of the destructive generalists of rice in Asia, southern Europe, and northern Africa^1–3^. The larvae bore into the rice stems and feed on tender tissues, resulting in ‘‘dead heart’’, ‘‘white heads’’ and ‘‘dead sheath’’ of the infested plants^4^. Thus severe yield and economic losses are caused per year, particularly in China due to the large cultivation of rice^3–8^. In recent years, an aquatic vegetable ‘water-oat’ is winning a warm praise from grower and customers, because of its economic and nutritional benefit^9^. It is grown year-round, facilitated by global warming, variety alternative, cropping system, and expansion of the greenhouse, thus increase the damage of this pest species. The intercropping pattern (rice is planted in a mosaic fashion under a crop rotation system with the water-oat) facilitates a transfer of *C. suppressalis* from rice plant to water-oat plant. Both the rice and water-oat belong to the tribe Oryzeae (family Gramineae) and possess the similar habitat, biology and ecology, but their nutrients and allelochemicals are markedly different^9–13^. Although *C. suppressalis* can complete their life cycles on rice or water-oat, those feeding on water-oat fruit pulps possess higher survival rate, pupal weight and shorter developmental duration than those feeding on rice^10,11,14–17^. After a long period of adaptation, *C. suppressalis* has diverged phenotypically into rice population and water-oat population^11,16,18–21^. The divergent populations exhibit significant phenotypic differences in morphology^10,15,16^, behaviors^11,19,20,22,23^, biochemical and physiological indexes^6,11,19,20,22–24^, emergence peak^14,15,25^, genetic differences^19,26^, ecological and biological characters^10,14,17,20,22,27^. Despite these variations were revealed after host shift, the mechanisms underlying host plant adaptation remains unclear.

The insects’ gut is a tube opening from the mouth to the anus, and is divided into three distinct regions, the foregut, midgut and hindgut. Food is usually stored in the foregut; useful materials (nutrients) are absorbed in the midgut, and partial nutrients and water are reabsorbed in the hindgut. The gut is a desirable, nutrient-rich ecological niche where multiple microbial taxa flourish and reproduce. The anterior hindgut is the most densely symbiont-inhabited site, due to the available, partially digested food being from the midgut, and the excretions from the Malpighian tubules^28^. The microbial taxa perform nutritional role to their hosts by providing nutrients lacking in hosts’ diets. Additionly, some species of the bacteria contribute to the other various functions, including immune, development, survival, reproduction, detoxification^29–36^ and population differentiation^37^. Therefore, they can help the insects adapt to host plants successfully. This is especially obvious in phytophagous insect, because of many secondary materials existed in host plants.

Over a long period of coevolution, a symbiosis has been formed between gut bacteria and their insect hosts. Gut bacteria exhibit some plasticity, and their communities alter with the change of insect diet^38,39^. Such an adaptiveness supplies a solid foundation for the development of host-associated differentiation^40^. Further, it has demonstrated that the population divergence is associated with microorganisms, and the symbiotic bacteria are important factors that promote evolution in the genus *Nasonia*^37^ and fruit fly *Drosophila melanogaster*^41^. Microbial community associated with insects feeding on alternative host plants were commonly reported in other lepidopterans^38,40,42–46^. However, the dynamics interactions between *C. suppressalis* populations and their gut microbiota are far from being understood, excepting for association of insecticides and gut bacteria^47,48^. Thus, a complete characterization of host-associated variation in bacteria composition is indispensable for an overall understanding of insect-bacteria symbioses of this species, and for the development of novel biocontrol management strategies. Herein, data from 16S rRNA next-generation amplicon sequencing were used, with the aiming to characterize the gut microbial communities of phenotypically divergent populations of larval *C. suppressalis*, as well as to explore ecological questions related to the host and gut compartment.

## Methods

### Specimen collection and rearing

In order to obtain representative populations of water-oat and rice, larvae of *C. suppressalis* were collected from the water-oat field in Lishui and the rice field in Yuyao, Zhejiang, China in 2018, where rice TN1 and water-oat *Zizania latifolia* are exclusively planted. During the experiment in our laboratory, rice-seedlings and water-oat pulps were collected from our institutional experimental field. The use of plant parts in the present study complies with institutional guidelines. The *C. suppressalis* larvae from the water-oat and rice fields were reared respectively with fresh water-oat fruit pulps and rice seedlings. *C. suppressalis* larvae were kept in an insectarium at 28 ± 1 °C, with a photoperiod of 16 h: 8 h (light/dark), and a relative humidity > 80%. All the larvae were maintained in the laboratory for three generations before dissection.

We analyzed the 16S rRNA gene to estimate the gut bacterial composition in the midgut and hindgut of larvae feeding on water-oat fruit pulps and rice seedlings. Treatments, abbreviations, and locations of the samples were provided in Table 1. *C. suppressalis* fed with their original hosts were named as original populations, and those fed with non-original hosts were named as cross-rearing populations.

**Table 1:** Sample list of larvae *C. suppressalis*.

Populations	Sample name	Diet type	Gut compartment	Location
**Original populations**	JMG	Water-oat fruit pulps	Midgut	Lishui, Zhejiang
	JHG	Water-oat fruit pulps	Hindgut	Lishui, Zhejiang
	RMG	Rice seedlings	Midgut	Yuyao
	RHG	Rice seedlings	Hindgut	Yuyao
**Cross-rearing populations**	jMG	Rice seedlings	Midgut	Lishui, Zhejiang
	jHG	Rice seedlings	Hindgut	Lishui, Zhejiang
	rMG	Water-oat fruit pulps	Midgut	Yuyao
	rHG	Water-oat fruit pulps	Hindgut	Yuyao

### Experimental design

**Figure 1:**
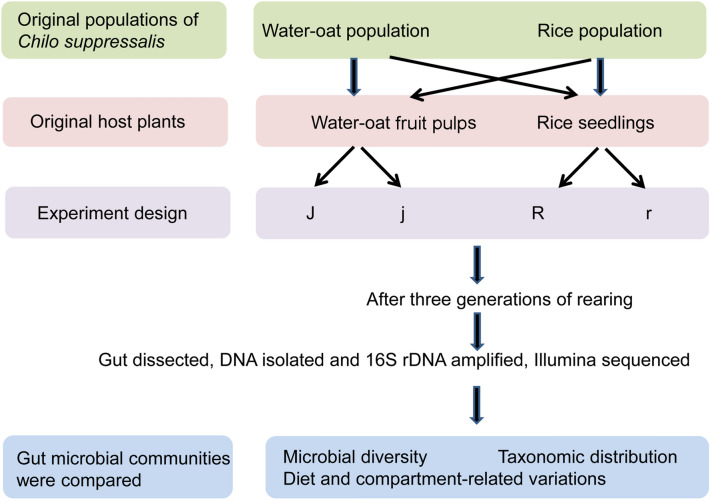
Schematic diagram of the fully factorial experimental design used in the present study. *Chilo suppressalis* from water-oat field and rice field were reared on water-oats fruit pulps or rice seedlings for three continuous generations to examine the effects of host plant and population origin on the gut microbiota. There were two cross-rearing populations (i.e., non-original populations) comprised of water-oat population individuals reared on rice seedlings (j) and rice population individuals reared on water-oat fruit pulps (r). The corresponding original populations were water-oat population individuals reared on water-oat fruit pulps (J) and rice population individuals reared on rice seedlings (R).

Schematic diagram of the fully factorial experimental design used in the present study. * C. suppressalis* from water-oat field were respectively reared on water-oat fruit pulps (J) and rice seedlings (j); and those from rice field were respectively reared on rice seedlings (R) and water-oat fruit pulps (r). All the groups were reared for three continuous generations ﻿ before examining the effects of host plant, population origin and gut compartment on the gut microbial communities (Fig. 1).

### *C. suppressalis* dissection and gut sample collection

Healthy, uniformly developed individuals of the same batch of *C. suppressalis* were collected*.* Each individual was anesthetized by placed on ice and externally sterilized with 75% ethanol and rinsed 3 times with sterilized water. The gut were dissected out with a sterilized fine-tip forcep and washed twice with sterile 0.9% NaCl solution quickly. The midgut and hindgut were carefully separated and placed in different sterile microcentrifuge tubes, synchronously. Midguts and hindguts of 50 individuals of each population were collected as one sample, and three samples were taken for each population. All samples were immediately frozen in liquid nitrogen and stored at − 80 °C for DNA isolation.

### DNA isolation, 16S rDNA amplification

Total bacterial genomic DNA was extracted from eight sets of sample groups using a Soil DNA Kit (Omega Bio-tek, Norcross, GA, U.S.) according to the manufacturer’s instructions. The DNA was finally eluted with TE buffer (Tris–EDTA buffer). DNA purity and concentration were measured using the NanoDrop 2000 spectrophotometer (Nano-drop Technologies, Wilmington, DE, USA). The total DNA was stored at − 70 °C until use.

The bacterial 16S rRNA variable V3−V4 regions were used to identify bacteria. Two universal primers (341F and 806R) containing the specific barcode sequence were used for the amplification of the V3−V4 regions (341F: 5’-CCTAYGGGRBGCASCAG-3’, 806R: 5’-GGACTACNNGGGTATCTAAT-3’). The Polymerase Chain Reaction (PCR) reaction was performed in triplicate 20.0 µL mixture containing 4.0 μL 5 × FastPfu Buffer, 2.0 μL 2.5 mM dNTPs, 0.8 μL of each Primer (5.0 μM), 0.4 μL FastPfu Polymerase, and 10 ng of template DNA. The amplification procedure was as follows: 95 °C for 2 min, followed by 25 cycles of denaturation at 95 °C for 30 s, annealing at 50 °C for 30 s, and elongation at 72 °C for 30 s and a final extension at 72 °C for 5 min.

### Illumina MiSeq sequencing

Amplicons were extracted from 2% agarose gels and purified using a AxyPrep DNA Gel Extraction Kit (Axygen Biosciences, Union City, CA, U.S.) following the manufacturer’s protocols and quantified using QuantiFluor™-ST (Promega, U.S.). Purified amplicons were pooled in equimolar and paired-end sequenced (2 × 250) on an Illumina Novaseq6000 platform according to the standard instructions.

### Processing of sequencing data

Raw fastq files were demultiplexed, quality-filtered using QIIME (version 1.17) with the following criteria: (i) The 250 bp reads were truncated at any site receiving an average quality score < 20 over a 10 bp sliding window, discarding the truncated reads that were shorter than 50 bp; (ii) exact barcode matching, 2 nucleotide mismatch in primer matching, reads containing ambiguous characters were removed; (iii) only sequences that overlap longer than 10 bp were assembled according to their overlap sequence. Reads which could not be assembled were discarded.

Operational Units (OTUs) were clustered using UPARSE (version 7.1 http://drive5.com/uparse/) and chimeric sequences were identified and removed using UCHIME. The phylogenetic affiliation of each 16S rRNA gene sequence was analyzed by RDP Classifier (http://rdp.cme.msu.edu/) against the silva (SSU115) 16S rRNA database.

## Results

### General structure of gut

**Figure 2:**
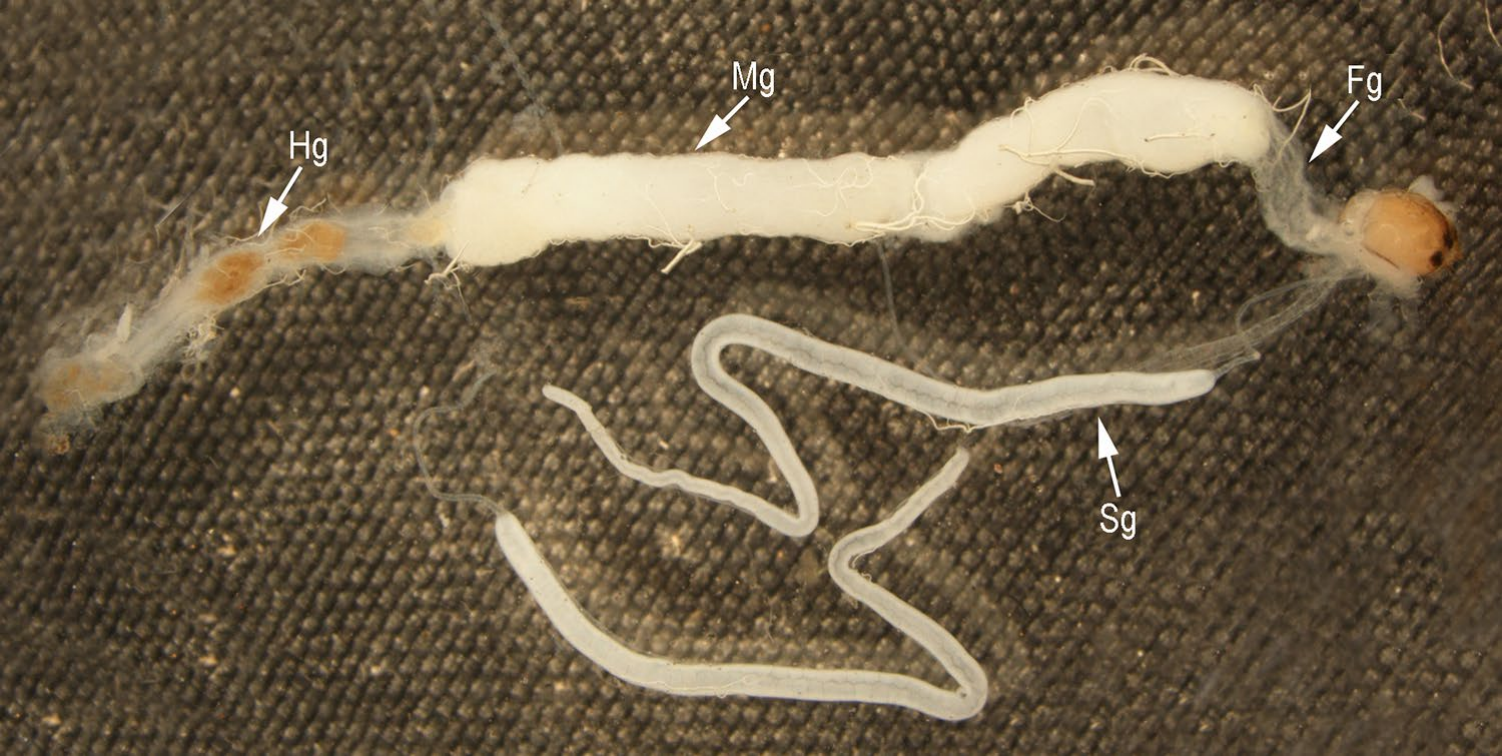
General structure of the gut and salivary glands of *Chilo suppressalis*. Fg, foregut; Mg, midgut; Hg, hindgut. Sg, salivary gland.

The gut of *C. suppressalis* was a continuous tube running from the mouth to the anus. It was structurally divided into foregut, midgut and hindgut. The foregut (Fg) was a slender, elongate tube, expanding posteriorly and constricting at its ends. The midgut (Mg) was a well-developed saclike tube beginning from the end of the foregut and extending to the long, narrow hindgut (Hg). The freshly dissected foregut was translucent, the midgut was opaque white, and the hindgut was yellowish-brown (Fig. 2).

### Analysis of bacterial 16S rDNA gene sequences

Illumina sequencing obtained 861,370 sequences clustering into 3234 operational taxonomic units (OTUs) (Table 2). Chao1 estimator and Shannon Index were calculated for the determination of the richness and homogeneity of the community. The relative bacterial abundance of 18 phyla differed significantly across the eight samples (*Kruskal–Wallis* test, *p* < 0.0001). The midgut and hindgut of the rice population feeding on water-oat fruit pulps (rMG, rHG), possessed the highest bacteria diversity. The bacteria in the midgut were more diverse than those in the hindgut (Table 2).

**Table 2:** Diversity of gut bacterial communities based on sequencing.

Insect populations	Diet type	Gut compartment	Abbreviations	Reads	Bases (bp)	OTUs	Coverage	Richness estimate		Diversity index
								Chao1	S	Shannon
**Original populations**	**WO**	**Midgut**	JMG1	40,138	17,434,180	248	0.998729	303	40,769	1.82
			JMG2	30,164	13,022,139	156	0.998110	225	30,361	1.76
			JMG3	38,225	16,566,823	185	0.998483	242	38,626	2.03
		**Hindgut**	JHG1	39,198	17,058,995	154	0.998954	182	39,778	1.78
			JHG2	29,999	13,129,341	97	0.999033	142	30,626	1.84
			JHG3	41,964	18,898,628	105	0.999261	129	44,068	1.45
	**RS**	**Midgut**	RMG1	29,622	13,457,815	116	0.999122	134	31,413	1.29
			RMG2	31,377	14,267,149	119	0.999076	140	33,296	1.31
		**Hindgut**	RHG1	42,226	18,171,436	94	0.999124	145	42,360	1.65
			RHG2	40,005	17,192,557	137	0.998700	178	40,081	0.78
			RHG3	36,054	15,537,581	100	0.998835	151	36,219	1.35
**Cross-rearing populations**	**WO**	**Midgut**	jMG1	38,043	16,419,021	60	0.999448	95	38,220	0.86
			jMG2	42,406	18,247,431	77	0.999245	148	42,501	1.00
			jMG3	37,306	16,100,611	66	0.999383	94	37,483	0.89
		**Hindgut**	jHG1	30,535	14,170,587	102	0.999247	115	33,125	1.85
			jHG2	41,371	18,295,563	106	0.999202	150	42,683	1.68
			jHG3	35,379	16,197,473	117	0.998954	159	37,890	1.94
	**RS**	**Midgut**	rMG1	42,873	18,879,467	209	0.998904	245	44,039	1.62
			rMG2	29,291	13,496,628	147	0.998669	196	31,641	2.02
			rMG3	30,888	14,254,538	277	0.997960	342	33,311	2.51
		**Hindgut**	rHG1	39,706	18,019,083	205	0.998187	290	42,137	2.43
			rHG2	37,724	16,820,944	174	0.998940	209	39,318	2.29
			rHG3	30,319	13,404,392	183	0.998450	232	31,425	2.81

*S* number of sequences, *WO* water-oat, *RS* rice seedlings.

### Microbial diversity of gut microbiota

**Figure 3:**
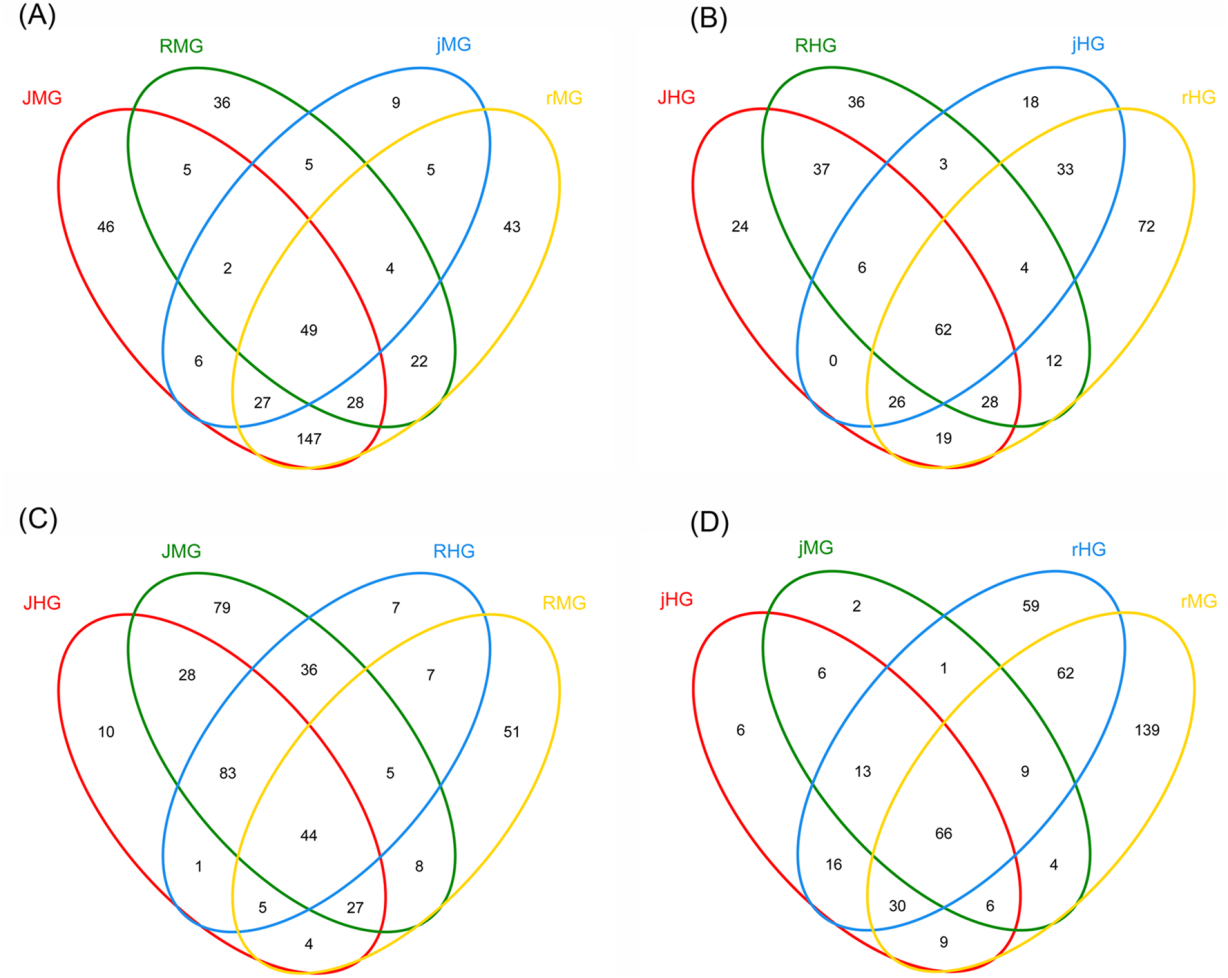
Venn diagram of OTUs from unique species owned by each sample and common species shared by two or more samples. (**A**) Midgut samples of cross-rearing populations and original populations. (**B**) Hindgut samples of cross-rearing populations and original populations. (**C**) Midgut and hindgut samples of original populations. (**D**) Midgut and hindgut samples of cross-rearing populations. *JMG* midguts of water-oat population feeding on water-oat fruit pulps, *RMG* midguts of rice population feeding on rice seedlings, *jMG* midguts of water-oat population feeding on rice seedlings, *rMG* midguts of rice population feeding on water-oat fruit pulps, *JHG* hindguts of water-oat population feeding on water-oat fruit pulps, *RHG* hindguts of rice population feeding on rice seedlings, *jHG* hindguts of water-oat population feeding on rice seedlings, *rHG* hindguts of rice population feeding on water-oat fruit pulps.

A total of 49 and 62 OTUs were observed in the midguts and hindguts, respectively (Fig. 3A,B). The core OTUs identified belonged to the phyla Proteobacteria, Firmicutes, Actinobacteria, Saccharibacteria, and Bacteroidetes (S1 Fig). The OTUs detected from the midgut were grouped into 31 families, of which five families were abundant: Enterobacteriaceae (24.6%), Halomonadaceae (20.2%), Enterococcaceae (31.4%), Bacillaceae (11.4%), and Streptococcaceae (6.9%) (S1A Table; S2 Table and S3 Table). However, the OTUs coming from the hindgut were grouped into 28 families. The predominent families were Enterobacteriaceae (66.4%), Enterococcaceae (11.2%), Bacillaceae (5.0%), Streptococcaceae (3.0%), Xanthomonadaceae (2.2%) and Flavobacteriaceae (1.7%) (S1B Table; S2 Table and S3 Table). The rMG and rHG had the maximum number of unique OTUs, whereas the midgut and hindgut of the water-oat population feeding on rice seedlings (jMG, jHG) possessed the minimum number.

A total of 44 and 66 OTUs were observed in the guts of original and cross-rearing populations, separately (Fig. 3C,D). These OTUs were pooled into 26 families for the midgut of original populations. The relative abundances of five families were Enterobacteriaceae, Halomonadaceae, Bacillaceae, Enterococcaceae and Streptococcaceae (S1C Table; S2 Table and S3 Table). However, in the hindgut of cross-rearing populations, the OTUs were grouped to 35 families, of which the abundant ones were Enterobacteriaceae, Enterococcaceae, Streptococcaceae, Xanthomonadaceae and Halomonadaceae (S1D Table; S2 Table and S3 Table).

### Taxonomic distribution of gut bacteria

**Figure 4:**
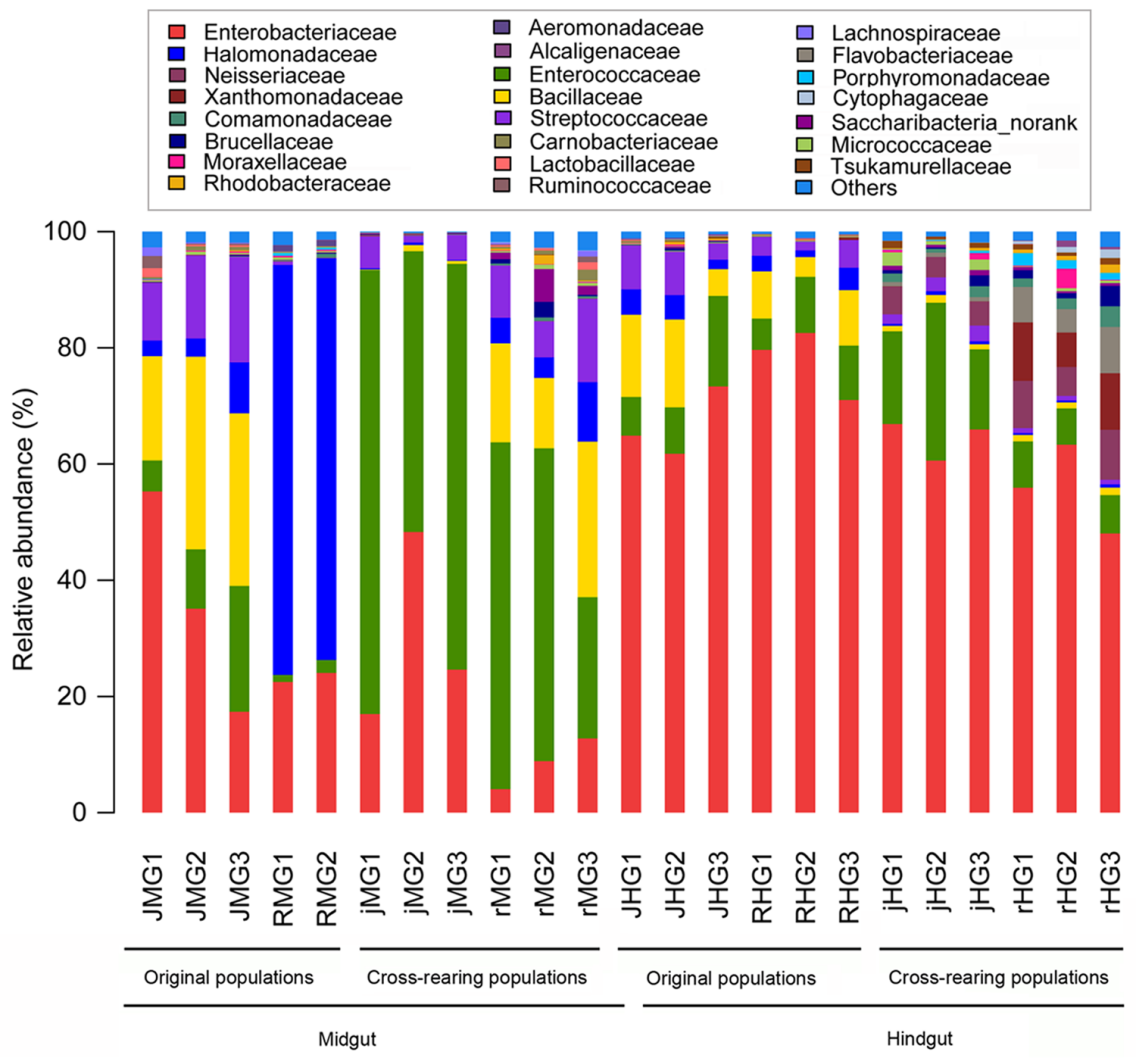
Compositions of gut microbiota at the family level of original and cross-rearing populations of *C. suppressalis*. The Y-axis represents the proportion of each taxon. *JMG1–JMG3* midguts of water-oat population feeding on water-oat fruit pulps, *RMG1–RMG2* midguts of rice population feeding on rice seedlings, *jMG1–jMG3* midguts of water-oat population feeding on rice seedlings, *rMG1–rMG3* midguts of rice population feeding on water-oat fruit pulps, *JHG1–JHG3* hindguts of water-oat population feeding on water-oat fruit pulps, *RHG1–RHG3* hindguts of rice population feeding on rice seedlings, *jHG1–jHG3* hindguts of water-oat population feeding on rice seedlings, *rHG1–rHG3* hindguts of rice population feeding on water-oat fruit pulps; Original populations: *C. suppressalis* collected from water-oat field and reared on water-oat fruit pulps; or *C. suppressalis* collected from rice field and reared on rice seedlings. Cross-rearing populations: *C. suppressalis* collected from water-oat field but reared on rice seedlings; or *C. suppressalis* collected from rice field but reared on water-oat fruit pulps. Abbreviations for each sample are explained in Tables 1 and 2.

Taxonomic classification yielded 122 families belonging to 18 bacterial phyla (Fig. 4; S3 Table and S4 Table), and the predominant phyla were Proteobacteria (16.0–96.4%), followed by Firmicutes (2.3–78.9%). At the family level, Enterobacteriaceae (8.0–78%) was the most predominant taxa, followed by Enterococcaceae (1.7–64.2%) and Halomonadaceae (0.3–69.8%) (S3 Table). The family Bacillaceae was only found in the water-oat population and two cross-rearing populations, although it was low relative abundance (Fig. 4; S3 Table). It was enriched in the midgut of the water-oat population (JMG ) (17.9–33.1%), followed by the midgut of the rice population feeding on water-oat fruit pulps (rMG) (17.0–26.8%) and the hindgut of the water-oat population (JHG) (4.6–15.1%). They exhibited a high variation of relative abundance associated with diet and gut compartment, though the most abundant taxa were identified in the midgut and hindgut of all populations.

Regardless of diet, a more homogeneous phylum distribution was found in the hindguts of all original populations and cross-rearing populations (i.e., hindguts of the water-oat population feeding on water-oat fruit pulps (JHG) and rice seedlings (jHG), hindguts of the rice population feeding on rice seedlings (RHG) and water-oat fruit pulps (rHG)): Proteobacteria (71.5–80.9%), Firmicutes (9.0–27.7%), Bacteroidetes (0.1–8.8%), Actinobacteria (0.1–2.8%) and Saccharibacteria (0.6%), respectively (Fig. 4; S1 Figure; S3 Table and S4 Table). However, community of the Firmicutes and Proteobacteria was changed in the midgut of the water-oat population feeding on water-oat fruit pulps (JMG) (40.3–71.8%, 27.3–58.6%), midgut of the water-oat population feeding on rice seedlings (jMG) (50.6–82.0%, 17.8–49.0%) and midgut of the rice population feeding on water-oat fruit pulps (rMG) (72.1–87.3%, 10.5–24.8%). Four bacterial phyla in the midgut of the rice population feeding on rice seedlings (RMG) were more homogeneous in richness: Proteobacteria (96.2–96.6%), Firmicutes (2.2–2.3%), Bacteroidetes (0.6–0.9%) and Actinobacteria (0.3–0.5%).

**Figure 5:**
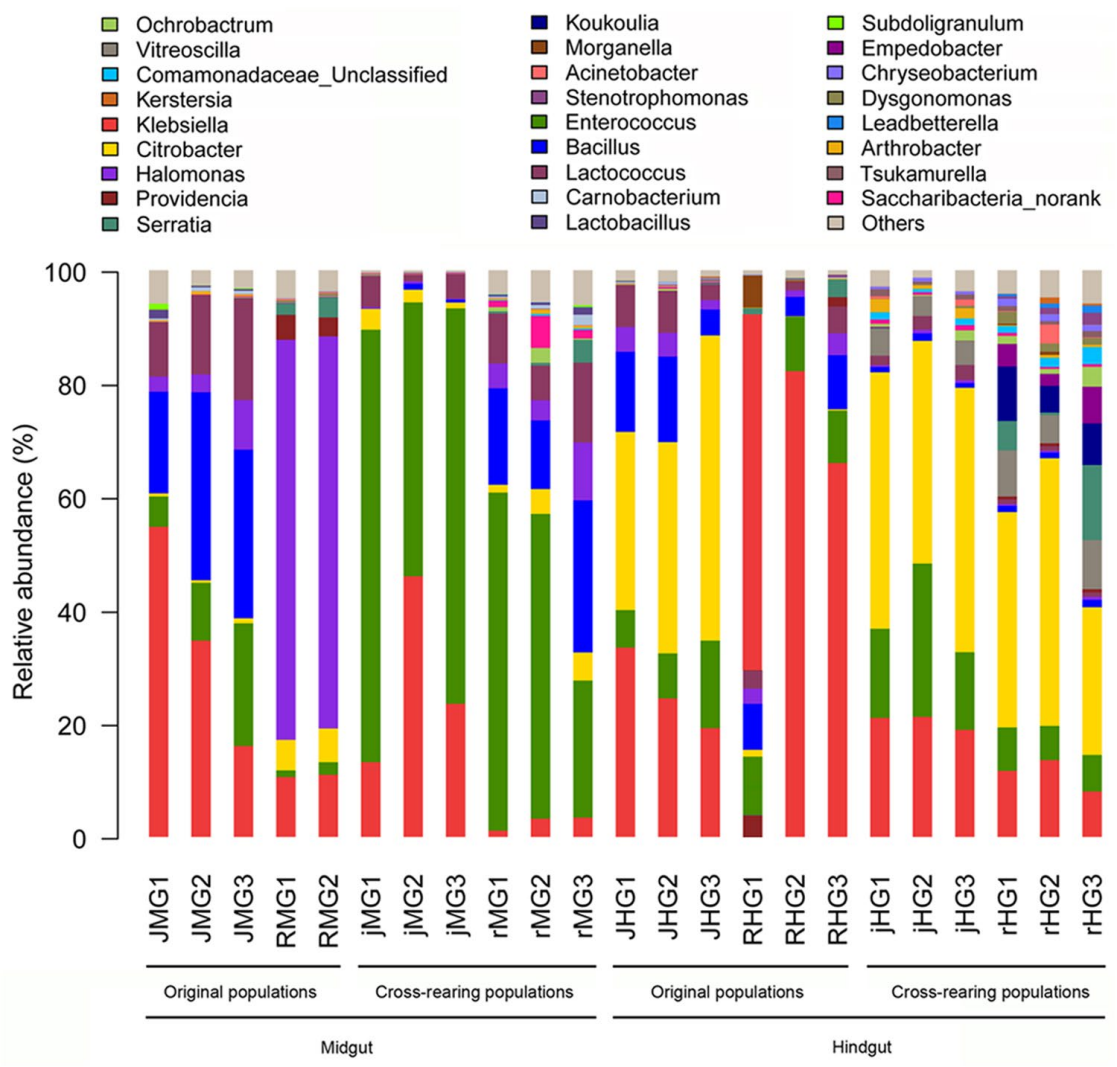
Compositions of gut microbiota at the genus level of original and cross-rearing populations of *C. suppressalis*. The composition of each sample was based on the taxonomic assignment of the 16S rDNA sequences. The Y-axis represented the proportion of each taxon. *JMG1–JMG3* midguts of the water-oat population feeding on water-oat fruit pulps, *RMG1–RMG2* midguts of rice population feeding on rice seedlings, *jMG1–jMG3* midguts of the water-oat population feeding on rice seedlings, *rMG1–rMG3* midguts of rice population feeding on water-oat fruit pulps, *JHG1–JHG3* hindguts of the water-oat population feeding on water-oat fruit pulps, *RHG1–RHG3* hindguts of rice population feeding on rice seedlings, *jHG1–jHG3* hindguts of the water-oat population feeding on rice seedlings, *rHG1–rHG3* hindguts of rice population feeding on water-oat fruit pulps; Original populations: *C. suppressalis* collected from water-oat field and reared on water-oat fruit pulps; or *C. suppressalis* collected from rice field and reared on rice seedlings. Cross-rearing populations: *C. suppressalis* collected from water-oat field but reared on rice seedlings; or *C. suppressalis* collected from rice field but reared on water-oat fruit pulps. Abbreviations for each sample are explained in Tables 1 and 2.

The bacterial genera from original populations showed distinct distribution according to diet types and gut compartments (Fig. 5; S5 Table). *Halomonas* (69.9%) and *Klebsiella* (70.1%) were dominant in the midgut and hindgut of the rice population feeding on rice seedlings (RMG and RHG); but *Bacillus* (26.9%) and *Klebsiella* (35.14%) were prevailed in the midgut of the water-oat population feeding on water-oat fruit pulps (JMG), *Citrobacter* (40.8%) was enriched in the hindgut of the water-oat population feeding on water-oat fruit pulps (JHG). *Enterococcus* was dominant in the midguts of the two cross-rearing populations (jMG (64.8%) and rMG (45.9%)), and *Citrobacter* was prevailed in the hindguts of the two cross-rearing populations (jHG (43.7%) and rHG (37.1%)). However, the bacteria in cross-rearing populations showed different genus distributions based on diet types. The *Klebsiella* (27.6%) and *Bacillus* (18.7%) were the relative dominance in jMG and rMG, whereas the *Enterococcus* (18.9%, 6.7%) and *Klebsiella* (20.4%, 11.1%) were relatively prevalent in jHG and rHG.

### Diet- and compartment-related variations in the gut microbial composition

**Figure 6:**
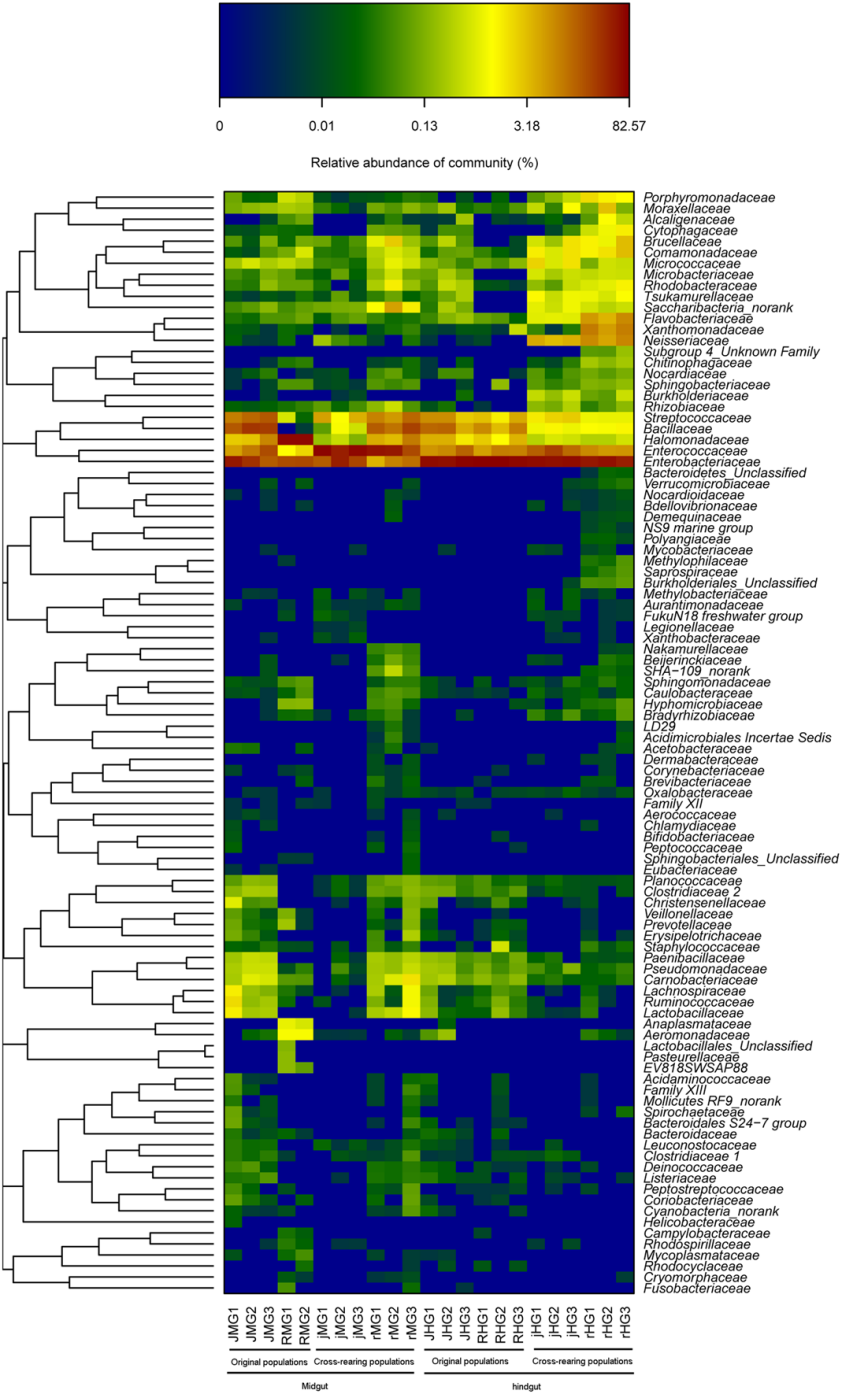
Abundance and composition of gut microbiota of all populations. Heatmap represent the proportions of OTUs at the family level. The X-coordinate represents the sample of each population, and the Y-coordinate represents the taxon. The color code indicates relative abundance, ranging from blue (low abundance) to yellow to red (high abundance). *JMG1–JMG3* midguts of the water-oat population feeding on water-oat fruit pulps, *RMG1–RMG2* midguts of rice population feeding on rice seedlings, *jMG1–jMG3* midguts of the water-oat population feeding on rice seedlings, *rMG1–rMG3* midguts of rice population feeding on water-oat fruit pulps, *JHG1–JHG3* hindguts of the water-oat population feeding on water-oat fruit pulps, *RHG1–RHG3* hindguts of rice population feeding on rice seedlings, *jHG1–jHG3* hindguts of the water-oat population feeding on rice seedlings, *rHG1–rHG3* hindguts of rice population feeding on water-oat fruit pulps; Original populations: *C. suppressalis* collected from water-oat field and reared on water-oat fruit pulps; or *C. suppressalis* collected from rice field and reared on rice seedlings. Cross-rearing populations: *C. suppressalis* collected from water-oat field but reared on rice seedlings; or *C. suppressalis* collected from rice field but reared on water-oat fruit pulps.

In all populations, there were significant differences in the relative abundances at the family level (*p* < 0.0001, *Kruskal–Wallis* test). 95 bacterial taxa were identified at the genus level. Influence of compartment sampling proved significant with a well-defined cluster formed by the midguts of all original and cross-rearing populations (i.e., JMG, jMG, rMG and RMG). By contrast, bacteria from the hindguts of all populations (i.e., RHG, JHG, jHG, rHG) were more heterogeneous for constituting four different clusters (Fig. 6). All the midguts and hindguts exhibited a significant difference in bacteria abundance of three families: Enterobacteriaceae, Enterococcaceae and Bacillaceae. Enterobacteriaceae was dominant in the hindgut (66.4%), but was decreased to 24.6% in the midgut. In comparison, Enterococcaceae was less abundant in the hindgut (11.2%), whereas increased to 31.4% in the midgut; Bacillaceae (5.0%) resided in the hindgut was increased to 11.4% in the midgut (Fig. 6; Table S3).

The differences at the family level were (Fig. 6): (1) a higher abundance of Enterobacteriaceae in the hindguts (55.8%) than in the midguts of the rice population feeding on water-oat fruit pulps (8.6%) and the water-oat population feeding on water-oat fruit pulps (35.9%); (2) a higher presence of Enterococcaceae in the midgut of the water-oat population feeding on rice seedlings (64.8%) than in the midgut and hindgut of the water-oat population feeding on water-oat fruit pulps (12.4%%, (10.0%), midgut of the rice population feeding on water-oat fruit pulps (45.9%) and the hindgut of the water-oat population feeding on rice seedlings (18.9%); (3) and a higher presence of Halomonadaceae in the midguts of two original populations (RMG: 69.9%; JMG: 4.9%) and the rice population feeding on water-oat fruit pulps (6.0%), hindguts of the water-oat population feeding on water-oat fruit pulps and rice seedlings (3.4%, 0.3%). However, the Bacillaceae was higher in the midgut and hindgut of the water-oat population feeding on water-oat fruit pulps (26.9%, 11.3%) and midgut of the rice population feeding on water-oat fruit pulps (18.6%), than that in the midgut of the water-oat population feeding on rice seedlings (0.6%) and hindgut of the rice population feeding on water-oat fruit pulps (1.2%).

**Figure 7:**
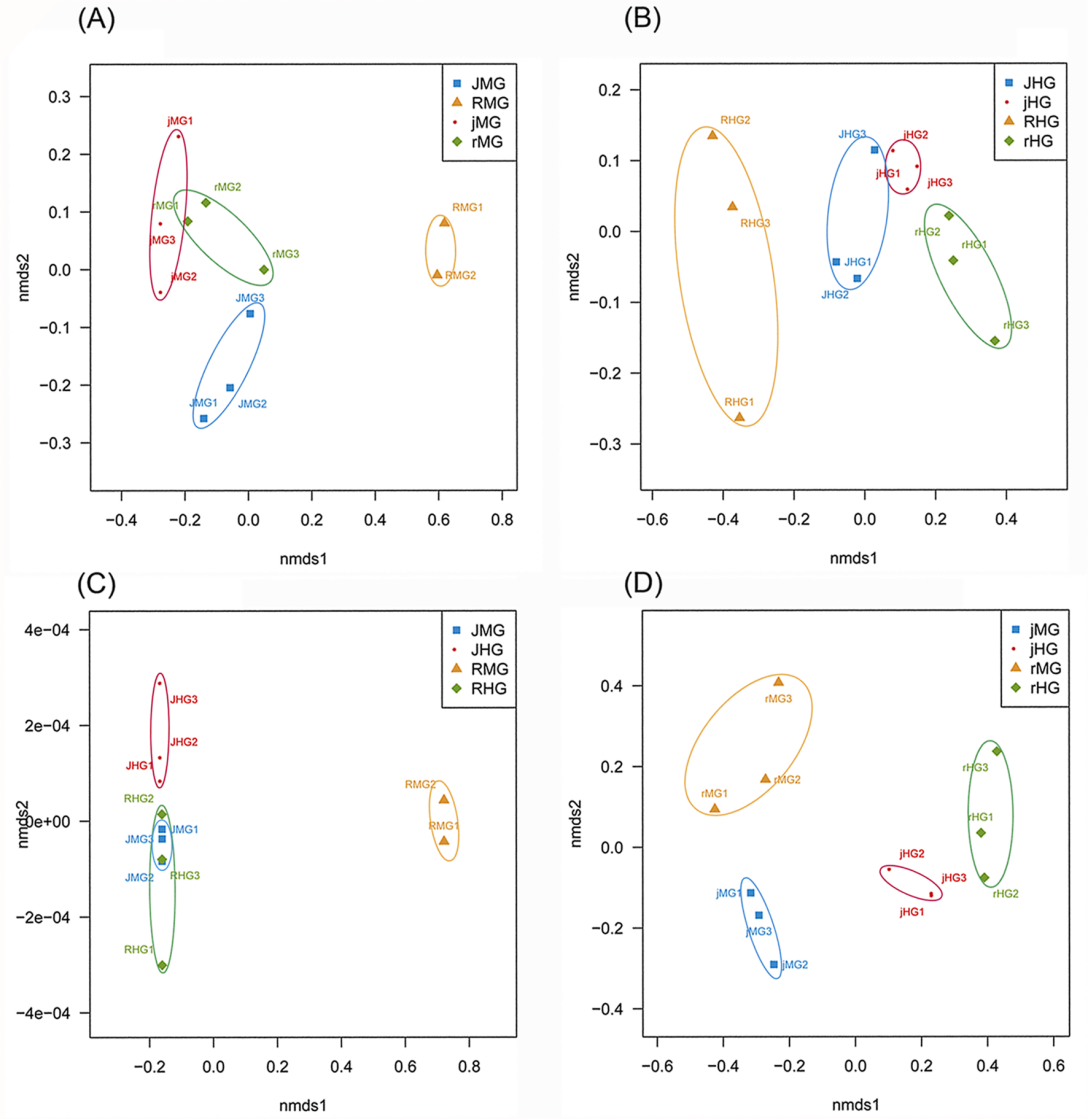
NMDS of the gut microbiota of *C. suppressalis*. (**A**) NMDS of the taxon distribution of midgut samples. The samples were clustered by diets and represented with different colors: jMG (red, circles), rMG (green, rhombus), JMG (blue, squares) and RMG (saffron yellow, triangle). (**B**) NMDS of the taxon distribution of the hindgut samples. The samples were clustered by diets and represented with different colors: jHG (red, circles), rHG (green, rhombus), JHG (blue, squares) and RHG (saffron yellow, triangle). (**C**) NMDS of the taxon distribution of the midgut and hindgut samples from the water-oat and rice populations. The samples were clustered by diets and represented with different colors: JHG (red, circles), RHG (green, rhombus), JMG (blue, squares) and RMG (saffron yellow, triangle). (**D**) NMDS of the taxon distribution of the midgut and hindgut samples from cross-rearing populations. The samples were clustered by diets and represented with different colors: jHG (red, circles), rHG (green, rhombus), jMG (blue, squares) and rMG (saffron yellow, triangle). The ellipses represent the standard error of the centroid for each group of samples with a confident limit of 95%.

A non-metric multidimensional scaling (NMDS) analysis was performed to analyze the influence of diet and compartment on the microbiota (Fig. 7A–D). The analysis revealed a clear separation of samples in accordance to the gut regions and a closer association among samples of the same gut region. At the midgut, the clusters were well defined and the highest variability was found in the RMG (i.e., midgut of rice population feeding on rice seedlings) cluster. The RMG and JMG (i.e., midgut of the water-oat population feeding on water-oat fruit pulps) clusters exhibited the most different taxa composition, followed by the rMG (i.e., midgut of the rice population feeding on water-oat fruit pulps) and jMG (i.e., midgut of the water-oat population feeding on rice seedlings) clusters, showing an intermediate composition (Fig. 7A). At the hindgut, there were clearly separated clusters: the RHG (i.e., hindgut of rice population feeding on rice seedlings) clusters exhibited a higher inter-sample variation; the JHG (i.e., hindgut of the water-oat population feeding on water-oat fruit pulps) cluster showed an intermediate composition respecting to the RHG, jHG (i.e., hindgut of the water-oat population feeding on rice seedlings) and rHG (i.e., hindgut of rice population feeding on water-oat fruit pulps) clusters (Fig. 7B). The midguts and hindguts clusters from all original populations (JMG, JHG, RMG, RHG) were well-defined, and the JMG, RHG clusters had similar homogeneity level (Fig. 7C). RMG was the most heterogeneous, followed by JMG and RHG. Clusters of cross-rearing populations were better defined than those of original populations. rMG was the most heterogeneous in taxa composition, followed by the jHG.

## Discussion

To date, there were few documents on how gut microbial communities differ across divergent insect populations based on diet and gut compartments. Gut bacterial diversity overall was notably greater in the rice population feeding on water-oat fruit pulps compared to the water-oat population or rice population feeding on rice seedlings. Bacterial communities resided in the midgut were more diverse and variable than those in the hindgut. Only bacteria of *Citrobacter, Enterococcus, Halomonas,* and *Klebsiella* were shared by original populations of *C. suppressalis*, and they were core microbiota based on their relative distribution*.* The core bacteria was able to colonize in different gut regions^49^, and might have evolved in closely related to hosts and were potential symbiont or beneficial bacteria^50–52^. Since rice seedlings and water-oat were very different in nutritional ingredient and secondary compounds, it was probable that the bacteria inhabited in *C. suppressalis* gut were beneficial to their hosts.

The gut bacterial composition and richness exhibited significant differences in the midgut and hindgut of different populations of *C. suppressalis*. *Halomonas* and *Klebsiella* dominated in the midgut and hindgut of rice seedlings-fed rice population, and *Klebsiella* and *Citrobacter* were prevailed in the midgut and hindgut of water-oat-fed water-oat population. *Enterococcus* were enriched in the midgut of cross-rearing populations, whereas *Citrobacter* was found exclusively in the hindgut of cross-rearing populations. According to Shao et al. And Zhang et al., the *Enterococcus* was associated with insecticide and pathogen resistances^53,54^, and the presence of this genus in *C. suppressalis* may enhance the immune of this pest during it host shift.

Our findings also showed that a remarkable different bacteria composition in the RMG and JMG and a intermediate bacteria composition in the rMG and jMG. The inter-individual variability was previously documented in honey bees *Apis mellifera*^55^, Anopheles^56^, and cockroaches *Blattella germanica*^38,57^, *Shelfordella lateralis*^58^ and *Periplaneta americana*^59^. The divergence in taxon composition may reflect divergent functional roles in specific resource use. Gut harbored bacteria community of the water-oat population and rice population feed on their original hosts is closely adapted to the *C. suppressalis’s* diets. With the change of diets, (i.e., water-oat population feeding on rice seedlings, and the rice population feeding on water-oat fruit pulps), the compositional change could be partly responsible for undergoing a recombination of the bacteria, accordingly. Curtis and Sloan suggested that the variation could be attributed to acquire microorganisms from a greatly diverse environmental reservoir microflora, randomly^60^. However, as the populations of *C. suppressalis* were reared many generations in identical laboratory conditions, such a variability could be ascribed to host genetics and population divergence, as was suggested by Sullam et al.^61^ Cluster analysis showed the jHG and rHG samples formed the most well-defined clusters, suggesting stable microbial profiles. The inter-individual differences suggested that SSB gut microbiome profiles may serve as useful biomarkers for bio-control in population-based studies.

The oligophagous diet of stem borers provided suitable ecological niches for harboring bacteria in compared with monophagous lepidopterans^55^. As the phyla Proteobacteria were reported to be involved in carbohydrate degradation, such as starches and hemicellulose^62^, and can be involved in pectin-degrading^63^ and nitrogen^64^. Firmicutes was suggested to take part in energy absorption from the diet and may influence the development^65^. The present results illuminated the abundance of two dominant phyla (i.e., Proteobacteria and Firmicutes) and the difference of three families (Enterobacteriaceae, Enterococcaceae and Halomonadaceae) in *C. suppressalis* populations. As the representative of the oligophagous, *C. suppressalis* feeding either on water-oat fruit pulps or rice seedlings. Both host plants shared the same family Gramineae, but their biochemical components and secondary substances were very different^9^. Our findings suggested that the rapid fluctuation of bacterial flora in larval gut was probably influenced by the biochemical components and secondary substances coming from the host plants; and the diet was an important factor in modulating the bacteria community, as was documented for other insect species^39,66–73^.

The gut bacterial genera were also varied, due to the difference of diets in *C. suppressalis*: in original populations, *Halomonas* was dominant in the RMG, *Klebsiella* was prevailed in the RHG and JMG, and *Citrobacter* was enriched in the JHG; in cross-rearing populations, *Enterococcus* was abundant in the midgut, and *Citrobacter* was predominant in the hindgut. Since diet and host taxonomy modulated bacteria community^71,74^, the successful expansion of bacteria over time probably in turn suppressed the bacteria growth from other phyla in the same habitat^66^. Therefore, we inferred that the different bacteria dominance might be related to successful reproduction of some bacteria genus and suppression of others. Whether the bacteria of *Citrobacter, Enterococcus, Halomonas,* and *Klebsiella* detected in the gut of original populations of *C. suppressalis* was truly associated with the host defense merits further investigation.

One interesting and unexpected result concerned the two compartments chosen for analysis, as we found that variability in microbial composition was higher in the midgut than in the hindgut, independently of diet. The obvious community difference indicated that only some specific groups of microorganisms were able to survive and colonize in the hindgut. However, Kacaniova et al. reported that the hindgut contained a higher number of anaerobic microorganisms than the midgut of honeybee^75^. Although the midgut and hindgut were alkaline, the unique morphology, favorable physiological conditions (viz., oxygen content, lack of unfavorable enzymes), and the availability of partially digested food could become a benign site for maintaining a special bacteria and quick proliferation in the hindgut of *C. suppressalis*. Indeed, this may be a controversial issue, and the different richness and colonization efficiency of the host symbiont indicated that further investigation should be done to understand their drivers.

## Conclusion

We investigated the gut microbial communities of two phenotypically divergent populations of *C. suppressalis*. The comparison of the midgut and hindgut microbia of *C. suppressalia* fed on the same diet provided insights into the compartment changes in the gut microbiota of SSB. Analysis of microbial community supplied an initial step toward improving our understanding of the mechanisms underlying *C. suppressalis* adaptation to host plants at the microbiological level. The results showed that the highest bacteria diversity was found for the midgut of the rice population feeding on water-oat fruit pulps. The most dominant phyla were Proteobacteria and Firmicutes; and the enriched families were Enterobacteriaceae, followed by Enterococcaceae and Halomonadaceae. The microbial communities were highly diverse at the genera level due to diet types or gut compartments among populations. The bacterial community composition was driven mainly by diet types, and affected by other factors including gut compartments. These findings provided an important insight into investigation of insect-bacteria symbioses, and biocontrol of this species and other lepidopterans.

## Supplementary Information

Supplementary Information 1.

Supplementary Information 2.

Supplementary Information 3.

## Data Availability

The raw reads were deposited into the National Center for Biotechnology Information (NCBI) Sequence Read Archive (SRA) database (accession no. SRP116573).

## Abbreviations

SSB: Striped stem borer
NMDS: Non-metric multidimensional scaling
PCR: Polymerase chain reaction
OTUs: Operational units
JMG: Midgut of water-oat population
JHG: Hindgut of water-oat population
RMG: Midgut of rice population
RHG: Hindgut of rice population
jMG: Midgut of water-oat population feeding on rice seedlings
jHG: Hindgut of water-oat population feeding on rice seedlings
rMG: Midgut of rice population feeding on water-oat fruit pulps
rHG: Hindgut of rice population feeding on water-oat fruit pulps
NCBI: National center for biotechnology information
SRA: Sequence read archive
