# Supplementary material for: Gut microbial communities associated with phenotypically divergent populations of the striped stem borer Chilo suppressalis (Walker, 1863)
Source: Sci Rep. 2021 Jul 22;11:15010. doi: 10.1038/s41598-021-94395-y (PMC8298391; doi:10.1038/s41598-021-94395-y)
Supplement: Supplementary file 2 — Supplementary Information 2. [file 41598_2021_94395_MOESM2_ESM.doc]

**Gut microbial communities associated with phenotypically divergent populations of the striped stem borer *Chilo suppressalis***

Haiying Zhong^1,2^, Juefeng Zhang^1,2^, Fang Li^1,2^ & Jianming Chen^1,2*^

^1^Institute of Plant Protection and Microbiology, Zhejiang Academy of Agricultural Sciences, Hangzhou, 310021, China

^2^State Key Laboratory for Managing Biotic and Chemical Threats to the Quality and Safety of Agro-products, Hangzhou 310021, China

*Corresponding. jianmchen63@163.com (Jianming Chen)

**
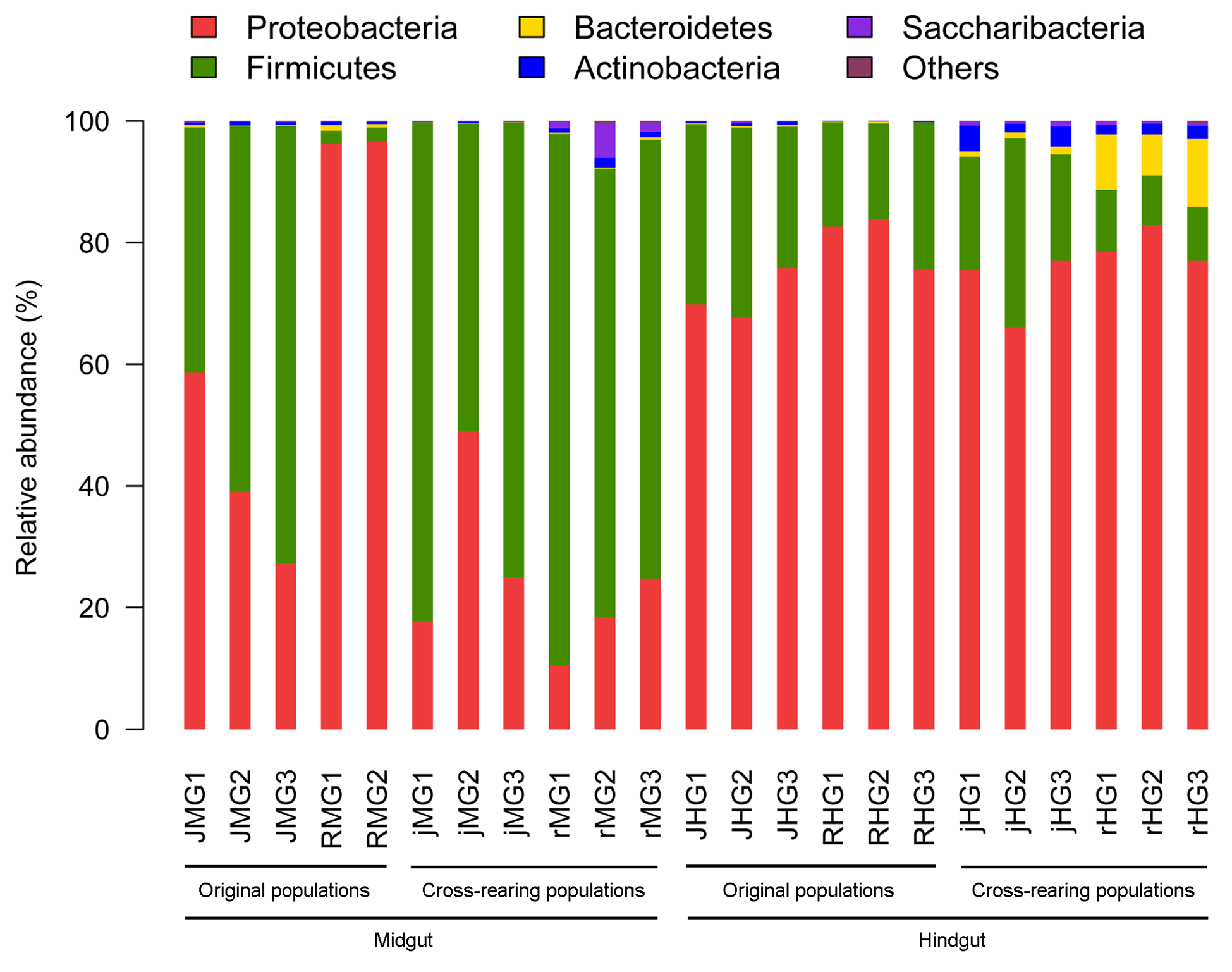
**

**Fig. S1.** Bacterial composition along the midgut and hindgut of original and cross-rearing populations. The composition of each sample was based on the taxonomic assignment of the 16S rDNA sequences. The Y-axis represented the proportion of each taxon [phylum and unclassified levels]. Abbreviations: JMG1–JMG3: midguts of the water-oat population feeding on water-oat fruit pulp; RMG1–RMG2: midguts of rice population feeding on rice seedlings; jMG1–jMG3: midguts of the water-oat population feeding on rice seedlings; rMG1–rMG3: midguts of rice population feeding on water-oat fruit pulp; JHG1–JHG3: hindguts of the water-oat population feeding on water-oat fruit pulp; RHG1–RHG3: hindguts of rice population feeding on rice seedlings; jHG1–jHG3: hindguts of the water-oat population feeding on rice seedlings; rHG1–rHG3: hindguts of rice population feeding on water-oat fruit pulp; Original populations: *C. suppressalis* collected from water-oat field and reared on water-oat fruit pulp; or *C. suppressalis* collected from rice field and reared on rice seedlings. Cross-rearing populations: *C. suppressalis* collected from water-oat field but reared on rice seedlings; or *C. suppressalis* collected from rice field but reared on water-oat fruit pulp.
