# Supplementary material for: Gut microbial communities associated with phenotypically divergent populations of the striped stem borer Chilo suppressalis (Walker, 1863)
Source: Sci Rep. 2021 Jul 22;11:15010. doi: 10.1038/s41598-021-94395-y (PMC8298391; doi:10.1038/s41598-021-94395-y)
Supplement: Supplementary file 3 — Supplementary Information 3. [file 41598_2021_94395_MOESM3_ESM.doc]

**Gut microbial communities associated with phenotypically divergent populations of the striped stem borer *Chilo suppressalis***

Haiying Zhong^1,2^, Juefeng Zhang^1,2^, Fang Li^1,2^ & Jianming Chen^1,2*^

^1^Institute of Plant Protection and Microbiology, Zhejiang Academy of Agricultural Sciences, Hangzhou, 310021, China

^2^State Key Laboratory for Managing Biotic and Chemical Threats to the Quality and Safety of Agro-products, Hangzhou 310021, China

*Corresponding. jianmchen63@163.com (Jianming Chen)

**Supplementary Table S1A: Please see the Excel file**

**Table S1A.** OUT name of bacteria of all midgut samples in venn.

**Supplementary Table S1B: Please see the Excel file**

**Table S1B.** OUT name of bacteria of all hindgut samples in venn.

**Supplementary Table S1C: Please see the Excel file**

**Table S1C.** OUT name of bacteria of midgut and hindgut samples of two original populations in venn.

**Supplementary Table S1D: Please see the Excel file**

**Table S1D.** OUT name of bacteria of midgut and hindgut samples of two cross-rearing populations in venn.

**Supplementary Table S2: Please see the Excel file**

**Table S2.** Classification table of bacteria species.

**Supplementary Table S3: Please see the Excel file**

**Table S3.** Mcrobial community percent at family level (unmerged biological replicates).

**Supplementary Table S4: Please see the Excel file**

**Table S4.** Mcrobial community percent at phylum level.

**Supplementary Table S5: Please see the Excel file**

**Table S5.** Mcrobial community percent at genus level.
